# Influence of Maternal Immunization with Allergens on the Thymic Maturation of Lymphocytes with Regulatory Potential in Children: A Broad Field for Further Exploration

**DOI:** 10.1155/2014/780386

**Published:** 2014-06-09

**Authors:** Jefferson Russo Victor

**Affiliations:** Laboratory of Dermatology and Immunodeficiencies, LIM-56, Medical School, University of Sao Paulo, Avenida Dr. Eneas de Carvalho Aguiar 500, Third Floor, 05403-000 Sao Paulo, SP, Brazil

## Abstract

A variety of mechanisms are involved in the regulation of offspring allergy development through maternal immunization with allergens. The passive transfer of antigens, antibodies, and cytokines, the induction of phenotypic alterations in offspring lymphocytes, and the induction of regulatory populations in offspring have been proposed, but these mechanisms remain incompletely understood. It is likely that maternal immunization could affect the intrathymic maturation of offspring TCD4+, TCD8+, *γδ*T, nTreg, iNKT, and B lymphocytes, although there are currently no human maternal immunization protocols for the regulation of allergic responses in children. Some studies have suggested a direct interaction between the maternal immune status and the offspring intrathymic microenvironment; this interaction could influence the maturation of offspring regulatory cells and must be explored for the development of therapies to control allergy development in children.

## 1. Introduction

In the last decade, we have studied the mechanisms involved in the regulation of offspring allergy development through maternal immunization with allergens. During this period, a variety of mechanisms have been studied, including the passive transfer of antigens, antibodies, and cytokines, the induction of phenotypic alterations in lymphocytes of offspring, and the induction of regulatory populations in offspring [1–6]. However, these findings have not fully elucidated the influence of maternal immunity. As reviewed in 2009, maternal murine immunization facilitates idiotypic interactions between maternal antibodies and the T-cell receptors (TCRs) of immature offspring T cells. This concept was introduced in the late 1980s when it was demonstrated that antibodies against TCRs could prevent intrathymic T cell maturation; however, this concept was not evaluated in the context of maternal immunization until recently [7]. Furthermore, other maternal factors, such as passively transferred cytokines, could influence the phenotype and maturation of offspring T cells [8]. Thus, it is likely that these interactions could affect the intrathymic maturation of offspring lymphocytes.

Currently, there are no human maternal immunization protocols for the regulation of allergic responses in children. However, a study of mothers undergoing immunotherapy for two years prior to gestation revealed that immunized mothers passively transferred high amounts of allergen-specific IgG antibodies to their children compared with nonimmune mothers [9]. Importantly, this feature was associated with a lower frequency of allergic diseases during the first two years of life in children from immunized mothers.

The thymus is the first lymphoid organ to form during pregnancy. At birth, the human thymus has approximately two-thirds of its mature weight and it reaches peak mass at approximately 10 years of age. The population of mature cells in the thymus is characterized by the expression of TCRs, comprising *α* and *β* chains, and the expression of CD4 and CD8 molecules. These cells reach maturity and migrate to secondary lymphoid organs, where several functions consistent with the effector and memory activity of the immune system are acquired. However, other populations of lymphocytes also mature in the thymus and can participate in the modulation/regulation of the immune system, as discussed below.

## 2. **α** **β**T Cells

*αβ*T cells represent approximately 95% of the mature T lymphocytes present in secondary lymphoid organs and peripheral blood. In response to antigen presentation, *αβ*TCD4+ (Th) cells can secrete large amounts of cytokines and *αβ*TCD8+ (Tc) cells acquire cytotoxic activity. Some Th cells can acquire natural regulatory properties, and this subset of cells will be discussed later (nTreg cells). The influence of maternal immunization upon the intrathymic maturation of these cells has not been described, but the effects of maternal HIV-infection have been evaluated. Thymi from children born to HIV-infected mothers are smaller in size compared to children born from noninfected mothers. Emigrant Th and Tc cells were evaluated phenotypically for the presence of T-cell receptor excision circles (TRECs), but no evidence of impaired thymic function or immune dysregulation was detected. Furthermore, the effects of the maternal antibody response to* Haemophilus influenzae* Type *b* vaccination were evaluated, and no effects were observed [10]. These results suggest that maternal infection or immunization cannot influence the *αβ*T cell thymic output of their children; however, until recently, these parameters were not evaluated in response to maternal immunization to allergens. It is possible that maternal cytokines produced in response to allergens can affect the child's thymus and modulate the Th cell cytokine profile or the cytotoxic activity of Tc cells. These responses could influence the Th1/Th2 balance of the children's T cells and induce other populations of cells, such as Th17 cells, after antigen stimuli. Furthermore, it has been shown that postnatal treatment of mice with antibodies against TCRs can prevent T cells maturation mainly by blocking the TCR-ligand interactions during intrathymic cell contact [7]. In this context, maternal antibodies produced in response to allergen immunization can be passively transferred and, after reaching the child's thymus, mediate some clonal selection of the T cell repertoire. As an indirect effect, the modulation/selection of T cells can influence the maturation/activation of regulatory cells in the thymus or secondary lymphoid organs. Taken together, these mechanisms can mediate the maternal influence on allergy induction and inflammation after allergen contact in children.

## 3. **γ** **δ**T Cells

*γδ*T cells represent approximately 5% of the mature T lymphocytes present in secondary lymphoid organs and peripheral blood. In humans, these cells can be observed at the fetal stage and confer immunity during the neonatal period before the full maturation of the adaptive immune system [11]. Primarily present in mucosal-associated lymphoid tissues (MALT), *γδ*T cells represent approximately half of the intraepithelial lymphocytes found in the intestine. The expression of TCRs comprising the *γ* and *δ* chains inhibits the interaction of these lymphocytes with MHC-peptide, in contrast to those that express receptors composed of *α* and *β* chains, including TCD4+ and TCD8+ cells [12]. Consequently, *γδ*T cells are not subject to the same selection and activation processes as *αβ*T cells. During maturation, murine *γδ*T cells express different phenotypes, and during the early stages of maturation, this population expresses the CD27, CD25, and CD24 molecules. Subsequently, CD25 expression is terminated, and only the combined expression of CD27 and CD44 is maintained [13].

However, *γδ*T cells express other membrane receptors that make them sensitive to the presence of cytokines, chemokines, and pathogen-associated molecular patterns (PAMPs). These ligands are responsible for the activation of *γδ*T cells and the subsequent induction of intense cytokine production. In mice, two major subpopulations of *γδ*T cells can be characterized according to IFN-*γ* or IL-17 production [14]. Phenotypically, these populations can be identified based on the expression of CD27, which is associated with IFN-*γ*-producing *γδ*T cells but not IL-17-producing *γδ*T cells. IL-17-producing *γδ*T cells have been implicated in the regulation of immunity against pathogens, such as* Salmonella enterica* and* Klebsiella pneumonia*, and in autoimmune diseases, such as experimental autoimmune encephalomyelitis (EAE) and chronic destructive arthritis [15–18].

IFN-*γ*-producing *γδ*T cells have been described in humans and can be stimulated via TCR using nonpeptide antigens, which results in *γδ*T enhanced cytotoxicity against a wide variety of tumor cells [19]. *γδ*T cells have become an interesting target for cancer immunotherapy [20], but these cells have not been considered targets for allergy treatment.

IL-17-producing *γδ*T cells have been identified in patients with psoriasis [21] and bacterial meningitis [22]. It has also been shown that IL-17-producing *γδ*T cells can ameliorate airway hyperresponsiveness and accelerate the resolution of eosinophilic inflammation in murine models of allergic airway inflammation [23]. Furthermore, it was recently shown that the adoptive transfer of non-antigen-specific *γδ*T cells from tolerized mice to OVA-immunized mice suppressed antigen-specific IgE production [24]. However, transgenic mice that overexpress Rae-1 specifically in keratinocytes produce higher levels of total serum IgE in response to the placement of ovalbumin patches on the skin when compared to control mice [25]. Rae-1 is a ligand of the NKG2D receptor, which is constitutively expressed on epithelial *γδ*T cells, raising the possibility that these cells can also contribute to the augmented IgE levels. These data support an important role for *γδ*T cells in allergy regulation. Until recently, the effect of maternal immunization through allergens or maternal atopy on the maturation, cytokine production, and function of offspring *γδ*T cells has not been evaluated. In particular, the effect of maternal immunization on *γδ*T cell maturation, particularly *γδ*T cells that produce IL-17, might represent a mechanism by which the maternal immune status could influence allergy development in children.

Furthermore, it was demonstrated that *γδ*T cells can exert regulatory functions on T cells and dendritic cells at tumor sites. V*δ*1 cell clones isolated and established from breast tumor TILs were responsible for this regulatory activity [26]. The origin of this cell population has not been elucidated, but another research group has shown that *γδ*T regulatory cells express the FOXP3 gene and are induced by the tumor microenvironment, suggesting that this cell population does not mature in the thymus [27].

It is possible that maternal cytokines produced in response to allergen immunization can reach the child's thymus and modulate the maturation of *γδ*T cells, particularly at the decision point between differentiating into IFN-*γ* or IL-17 *γδ*T producing cells, as regulatory T cells do not seem to be produced in the thymus.

## 4. Natural Regulatory T Lymphocytes (nTreg)

Characterized by the expression of *αβ* TCRs, CD4, and CD25 (IL-2R), nTreg cells represent 5–10% of the peripheral CD4+ T cells in both mice and humans [28]. These cells also express the transcription factor FOXP3 [29] and are characterized as CD4+CD25+FOXP3+ cells. These cells have potent regulatory functions and are naturally produced in the thymus [30]. In addition, these lymphocytes also express other membrane molecules, such as CD62 L, CTLA-4, CD28, GITR (TNF receptor-induced glucocorticoid), and neuropilin-1 [31]. A previous study showed that neonatal thymectomy in normal female mice aged 2 to 4 days resulted in immune deregulation, including ovarian autoimmune disease accompanied by inflammatory tissue damage in other organs and the detection of autoantibodies in the circulation. Indeed, thymectomized mice still developed thyroiditis, gastritis, orchitis, prostatitis, and sialadenitis [32]. These results could be interpreted as evidence of the importance of thymic nTreg cells on the maintenance of peripheral self-tolerance [33]. In humans, nTreg cells are matured at Hassall corpuscles in the thymus [31], and this process requires high-affinity interactions between TCRs and HLA-self peptides presented by thymic stromal cells [34] that modulate the maturation of nTreg cells. Furthermore, nTreg maturation requires costimulatory signals in the form of cytokines, such as IL-2 and IL-7, which are critical for the development of these cells [31]. This microenvironment also depends on thymic stromal lymphopoietin (TSLP) production, which strongly activates thymic dendritic cells (tDCs), inducing the overexpression of CD80 and CD86. Furthermore, tDCs express CD70, a CD27 ligand that is expressed on nTregs during thymic maturation. The CD27-CD70 interaction does not affect the functional differentiation of nTreg cells but can prevent their apoptosis [35]. The passive transfer of maternal cytokines can alter the phenotypic properties of tDCs and even direct interactions with immature nTreg cells, thereby modulating the function/maturation of these cells. A recent study showed that atopic mothers influenced the maturation of thymic nTreg cells in their children [36]. The suppressive capacity of thymic nTreg cells in children from atopic mothers is similar to that of nTreg cells from children of nonatopic mothers during the first 6 months of life, although this suppression is reduced at 5 years of age in children from atopic mothers, suggesting that maternal atopy induces a delay in the functional maturation of thymic nTreg cells. The involvement of nTreg lymphocytes in controlling offspring allergic responses through maternal immunization has previously been suggested based on the results obtained from a murine model of maternal immunization using allergens [2]. Subsequently, it was shown that nTreg cells could be induced through maternal sensitization using OVA, which was associated with offspring asthma control [37] and the induction of oral tolerance in offspring [38]. The results of these studies reveal the influence of the maternal immune status on the maturation of regulatory lymphocytes, which could influence allergy development in children. However, to date, the effect of maternal immunization through allergens or maternal atopy on the maturation, cytokine production, and lymphocyte function of thymic nTreg cells in neonates remains unknown.

## 5. Invariant NK T (iNKT) Cells

Murine iNKT cells are characterized by the expression of receptors comprising an *α* invariant chain (V*α*14J*α*18 and V*α*24J*α*18) and a *β* chain with low diversity. These lymphocytes recognize glycolipid antigens based on the membrane expression of the CD1d receptor, and a corresponding population has been identified in humans. iNKT cells produce high amounts of IL-4 and IFN-*γ* in response to *α*-glycolipid galactosylceramide, which promotes allergic lung inflammation [39] and increased susceptibility to pulmonary infections with* Listeria monocytogenes* [40].

It has also been recently shown that mice deficient in iNKT cells produce low levels of anaphylactic antibodies in response to allergy induced with Brazil nuts, and the activation of this population in normal mice is dependent on the presence of a lipid fraction of the allergen. In addition, this study also demonstrated that cells from atopic individuals produce IL-4 in response to allergen binding in a CD1d-dependent manner, suggesting that a similar mechanism occurs in humans [41].

Unlike CD4+ T cells that produce IL-17 (Th17), some iNKT cells can produce high amounts of IL-17 independent of the presence of IL-6 [42] but dependent on the presence of other cytokines such as IL-18 [43]. In mice, this iNKT IL-17+ subpopulation matures in the thymus through a different mechanism that is dependent on the expression of the retinoic acid receptor-related orphan receptor *γ*T (ROR-*γ*T). In addition, these lymphocytes do not express the NK1.1 receptor and produce high levels of IL-17 [44]. iNKT IL-17+ cells are primarily present in the lungs and are associated with neutrophil infiltration, as demonstrated in experimental animal models with impaired iNKT cells that show the reduced infiltration of neutrophils in response to the intranasal administration of *α*-glycolipid galactosylceramide [45].

Recently, it was shown that iNKT cells can exert some regulatory functions on TCD8+ cells in a murine model of allergic contact dermatitis. These iNKT cells were not characterized by the expression of membrane molecules, but it was shown that the activation of these cells is CD1d-dependent. Furthermore, the functional activity of these cells is mediated by IL-4 and IL-13 secretion and does not involve nTreg cell activation [46].

However, the effects of maternal immunization with allergens on the maturation and function of iNKT cells and subpopulations in newborns have not been described. As previously discussed, maternal cytokines can influence thymic maturation, including that of iNKT cells, which therefore represents an interesting field for further exploration.

## 6. B Cells

Although the process of thymic maturation results in the maturation of T lymphocytes and T cell subpopulations, lymphoid progenitors can induce B cell maturation in this organ, and this mechanism is apparently dependent on Notch signaling [47]. Indeed, the presence of B cell progenitors in the thymus of mice was described a decade ago [48]. Subsequently, phycoerythrin staining demonstrated that approximately 3 × 10^4^ cells/day emigrate from the adult mice thymus, showing a T cell-unrelated phenotype. These emigrant cells express IgM and B220 molecules and are therefore characterized as mature B cells [49]. In the same study, it was demonstrated that the majority of thymic B cells matured in the thymus, and only a minority of this population constituted infiltrated B cells. Cells in the primary stages of B cell maturation (pre-B and pro-B) have been identified among total thymocytes. Furthermore, blocking the maturation of T lymphocytes through TCR *β* chain gene inactivation increases the relative and absolute number of mature B cells in the thymus. However, the function of thymic B cells is not well understood. The participation of B cells in the negative selection of T cells has been demonstrated in a murine model of transgenic mice, in which B cells express MHC-II molecules specific to MOG peptides and T cells express specific MOG TCR receptors [50]. Furthermore, B cell-deficient mice revealed that the expression of tissue-restricted antigens, such as the MOG protein from thymic medullar epithelial cells (mtec), depended on the presence of B cells [51]. This study also suggested that this effect was mediated through the production of lymphotoxin (LT) in B cells. Taken together, these results suggest a direct participation of B cells in the shaping of the T and nTreg cell repertoires. It was also demonstrated that intrathymic B cells spontaneously produced IgA and IgG in greater quantities compared with splenic B cells, which produced IgM, in the absence of a predominant antigen-specific stimulus in a porcine model [52]. Taken together, these observations suggest that intrathymic B cells play different roles in immune regulation compared with splenic B cells.

Some subpopulations of B cells, such as B10 cells, exhibit regulatory potential upon allergy development [53], and populations of regulatory B cells (Breg) with high regulatory function have been described in both humans and mice. These cells can be phenotypically characterized as CD19+IL10+CD1d^high^  and produce high amounts of IL-10, and the regulatory potential of these cells has been demonstrated in terms of their inhibition of T cell-mediated [54] and allergic pulmonary inflammation induced by OVA in murine models [55]. It has also been reported that B10 lymphocytes participate in the regulation of Th17 and Treg cells during the development of murine autoimmune disease [56]. Moreover, the origin of B10 cells has not been described, and the thymic maturation of B10 cells represents an unprecedented hypothesis; however, it is possible that the thymus could play a role in this process. Thus, the mechanisms discussed above, including maternal passive transfer of antibodies that can interact with clonal receptors expressed at immature stages and cytokines that can modulate cell maturation and cytokine profiles, could directly influence B and/or Breg intrathymic maturation.

## 7. Conclusions

The results reported in the present study suggest that several populations of lymphocytes that can modulate or exert regulatory functions are matured in the thymus. Some of these cells exhibit an important role in allergy development, and the maturation of these cells can be influenced through cytokine modulation and idiotypic interactions during early immature stages. Thus, maternal immunization could indeed influence and directly impact allergy development in children, and the evaluation of this effect might yield an important perspective for the development of allergy regulation therapies.
